# Zero-crosstalk silicon photonic refractive index sensor with subwavelength gratings

**DOI:** 10.1186/s40580-024-00446-1

**Published:** 2024-09-28

**Authors:** Syed Z. Ahmed, Mehedi Hasan, Kyungtae Kim, Sangsik Kim

**Affiliations:** 1 Department of Electrical and Computer Engineering, Texas Tech University, Lubbock, TX 79409 USA; 2 School of Electrical Engineering, Korea Advanced Institute of Science and Technology, Daejeon, 34141 South Korea

**Keywords:** Silicon photonics, Refractive index sensor, Subwavelength grating, Metamaterials

## Abstract

Silicon photonic index sensors have received significant attention for label-free bio and gas-sensing applications, offering cost-effective and scalable solutions. Here, we introduce an ultra-compact silicon photonic refractive index sensor that leverages zero-crosstalk singularity responses enabled by subwavelength gratings. The subwavelength gratings are precisely engineered to achieve an anisotropic perturbation-led zero-crosstalk, resulting in a single transmission dip singularity in the spectrum that is independent of device length. The sensor is optimized for the transverse magnetic mode operation, where the subwavelength gratings are arranged perpendicular to the propagation direction to support a leaky-like mode and maximize the evanescent field interaction with the analyte space. Experimental results demonstrate a high wavelength sensitivity of − 410 nm/RIU and an intensity sensitivity of 395 dB/RIU, with a compact device footprint of approximately 82.8 μm^2^. Distinct from other resonant and interferometric sensors, our approach provides an FSR-free single-dip spectral response on a small device footprint, overcoming common challenges faced by traditional sensors, such as signal/phase ambiguity, sensitivity fading, limited detection range, and the necessity for large device footprints. This makes our sensor ideal for simplified intensity interrogation. The proposed sensor holds promise for a range of on-chip refractive index sensing applications, from gas to biochemical detection, representing a significant step towards efficient and miniaturized photonic sensing solutions.

**Graphical Abstract:**

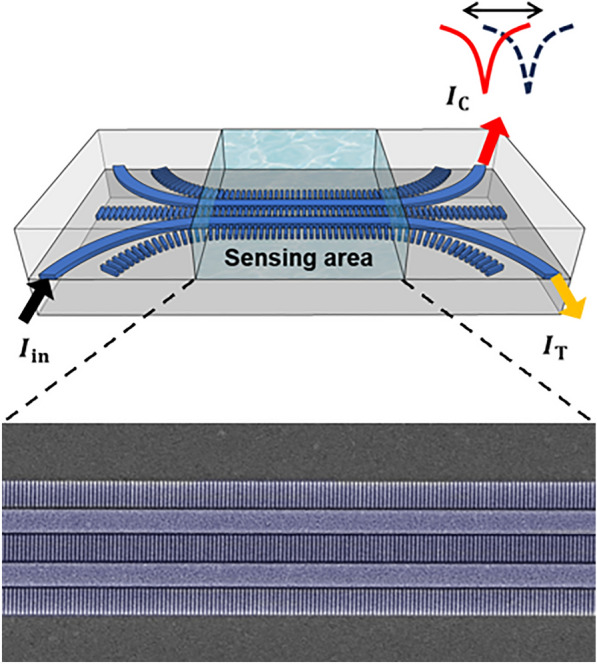

**Supplementary Information:**

The online version contains supplementary material available at 10.1186/s40580-024-00446-1.

## Introduction

Silicon photonics has emerged as a transformative technology for various optical applications, ranging from optical communication [1, 2], AI [3], quantum technology [4], and imaging system [5], leveraging the well-established CMOS foundry’s cost-effective and scalable production capabilities. While diverse sensing mechanisms exist involving light and nanostructures [6–8], silicon photonic approach is notable due to its small mode volume and tailorable evanescent wave interactions with variations in the cladding environment [9–24]. Silicon photonic refractive index (RI) sensors are particularly well-suited for label-free biosensing, offering a cost-effective and portable platform for point-of-care and lab-on-a-chip diagnostic tools [9, 10]. Various RI sensor configurations have been developed, typically based on interferometric or resonant structures, including Mach–Zehnder interferometer (MZI) [11–13], ring-resonator [14–17], cascaded MZI-ring [18–20], Bragg grating [21, 22], and photonic crystal (PhC) [23, 24]. Each scheme has strengths and limitations depending on its sensing mechanisms.

Interferometric sensors like MZI, for example, generally utilize cladding index variation to cause a phase shift that can be translated into either a wavelength shift or intensity variation. However, due to their sinusoidal spectral response, they suffer from ambiguous phase estimation and sensitivity fading near the maxima and minima [25, 26]. Solutions like phase modulation can address these issues [27, 28], but add complexity to the sensor architecture and cost. Moreover, MZI arms tend to be long to ensure sufficient interaction with analytes, leading to increased device footprints. On the other hand, resonant sensors like ring resonators translate cladding index variation to the resonant wavelength shift. These sensors can offer promising multiplexing capabilities with a large array of bioassays, which have even been commercialized by Genalyte Inc. [29, 30], but their need for expensive tunable lasers and their limited detection range due to finite free spectral range (FSR) pose challenges [31]. Another sensing scheme is a directional coupler (DC), which uses evanescent wave interaction to transfer optical power between closely spaced waveguides. They have a simple architecture and potential for intensity interrogation with a fixed wavelength and enhanced sensitivity. However, typical strip waveguides require a large device length to convert the cladding index difference-induced phase change into the output intensity variation [32, 33], and directional ambiguity can arise in calibrating the RI sensors, making them difficult to use in practice [34].

Recently, subwavelength grating (SWG) metamaterials have been widely used for silicon photonic RI sensors due to their ability to control the effective index of a waveguide. By engineering the filling fractions of SWGs, one can reduce the effective index of the guided mode, reducing the modal confinement and expanding the evanescent field into the analyte space, which could lead to a higher sensitivity. SWGs have been applied in various sensing schemes, including ring-resonators [35, 36], PhC [37, 38], Bragg [39] and bimodal waveguide [40] configurations. SWGs also exhibit highly anisotropic properties that can be used to engineer the skin depth and reduce crosstalk between closely spaced coupled waveguides [41, 42]. When two or more waveguides are coupled, SWGs can also be engineered to achieve zero-crosstalk at a specific parameter condition, utilizing anisotropic perturbation via SWGs [42, 43]. The coupled SWG waveguides scheme looks similar to a DC, but the zero crosstalk singularity is opposite to the directional coupling, having zero-crosstalk instead of complete power transfer. Since the zero-crosstalk condition is sensitive to the geometric parameters, it is intrinsically narrowband and sensitive to a local environment like the upper cladding index. Compared to other interferometric approaches like MZI, ring-resonator, and DC, the zero-crosstalk singularity exhibits a single transmission dip in the spectrum, free from the detection range limit due to nearby transmission dips. Furthermore, since the zero-crosstalk response is based on waveguide modal perturbation, it is independent of device length, while a DC response is significantly dependent on the device’s length [44]. This can eliminate the drawbacks of DC-based RI sensors, such as the large footprint and directional ambiguity, while retaining their advantages of simplicity, fixed wavelength intensity interrogation, and high sensitivity. The zero-crosstalk response can be achieved with either TE or TM modes, but for sensing applications, the TM zero-crosstalk response should be ideal since it has an extended evanescent field overlap with a sensing analyte, potentially achieving a higher sensitivity than the TE case.

In this paper, we introduce a silicon photonic RI sensor based on a coupled SWG waveguide scheme that utilizes an anisotropic perturbation-led zero-crosstalk response. We optimized the SWG waveguides for zero-crosstalk singularity with TM mode, which enables a single transmission dip. The SWGs are arranged perpendicular to the propagation direction, reducing the field confinement and having a large evanescent field gradient toward the sensing space. The single spectral response allows wavelength interrogation, circumventing the ambiguity issue that arises in typical MZI, ring-resonator, and DC-based sensors. The tangential nature of the spectral response allows an easier intensity interrogation scheme for a large RI range at a fixed wavelength, thus rendering this sensor architecture suitable for both wavelength and intensity sensing. Due to its FSR-free spectral response, the detection range is not limited by nearby FSRs but by the wavelength tuning range (i.e., in our setup, tunable laser source). Experimental investigations were conducted to characterize the device sensitivity and system limit of detection for both wavelength and intensity interrogations. Real-time sensing experiments were also conducted where a power change was detected as the cladding index gradually changed.

## Design and principle

**Fig. 1:**
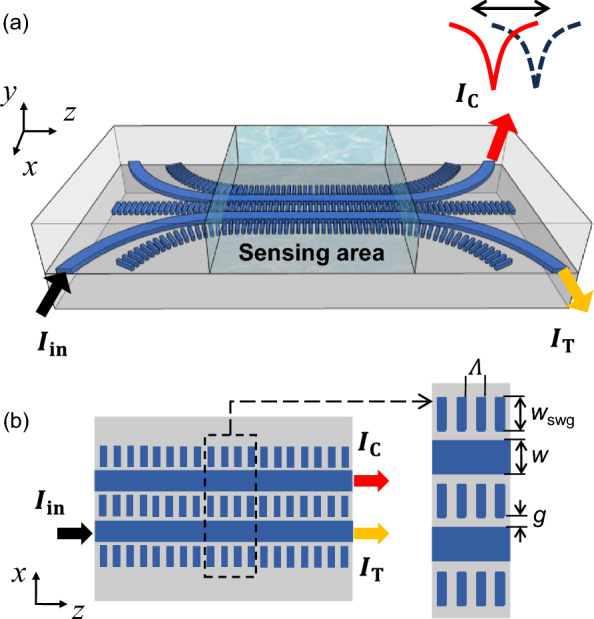
Schematic of the SWG-assisted zero-crosstalk RI sensor: **a** perspective, **b** top view. The blue and grey indicate Si core and $\hbox {SiO}_2$ cladding, respectively. The black, yellow, and red arrows indicate the input, through, and coupled ports, respectively. Typical parameters are $\Lambda =100$ nm, $\rho =0.5$, $\textit{w}=500$ nm, $\textit{w}_{\rm swg}=500$ nm, and $\textit{g}=65$ nm, unless otherwise specified

Figure 1 illustrates the schematic of the proposed RI sensor, comprising two coupled waveguides with SWGs in the cladding. The SWGs are arrayed in the propagation direction of the waveguide (*z*-axis), as shown in (a) perspective and (b) top views. The device is designed on a 220 nm thick silicon-on-insulator (SOI) platform, which is readily manufacturable with widely available fabrication processes. The geometric parameters are indicated in Fig. 1b: waveguide width $\textit{w}$, SWG width $\textit{w}_{\text{swg}}$, gap between waveguide and SWG $\textit{g}$, and periodicity of the SWG $\Lambda$. The black arrow indicates the power input ($\textit{I}_{\text{in}}$) to one of the waveguides, while the yellow and red arrows show the output powers in the through port ($\textit{I}_{\text{T}}$) and in the coupled port ($\textit{I}_{\text{C}}$), respectively. In the proposed RI sensor, sensing is achieved by monitoring the crosstalk spectrum changes at the coupled port due to index variations in cladding.

### Zero-crosstalk with the coupled SWG waveguides

To design and analyze the coupled SWG waveguides for achieving zero-crosstalk singularity, we employed effective medium theory (EMT) to model an anisotropic SWG metamaterials with permittivities $\varepsilon _x=\varepsilon _y=\varepsilon _\parallel$ and $\varepsilon _z=\varepsilon _\perp$ followed by [45],

1a $$\begin{aligned} \varepsilon _{\parallel }&=\rho \varepsilon _{\text{Si}}+(1-\rho )\varepsilon _{\text{clad}} \end{aligned}$$

1b $$\begin{aligned} \varepsilon _{\perp }&=\frac{\varepsilon _{\text{Si}}\varepsilon _\text{clad}}{\rho \varepsilon _{\text{clad}}+(1-\rho )\varepsilon _{\text{Si}}} \end{aligned}$$

where $\varepsilon _{\text{Si}}$ and $\varepsilon _{\text{clad}}$ are the permittivities of Si and cladding, respectively, and $\rho$ is the filling fraction of silicon (Si) in the cladding.

**Fig. 2:**
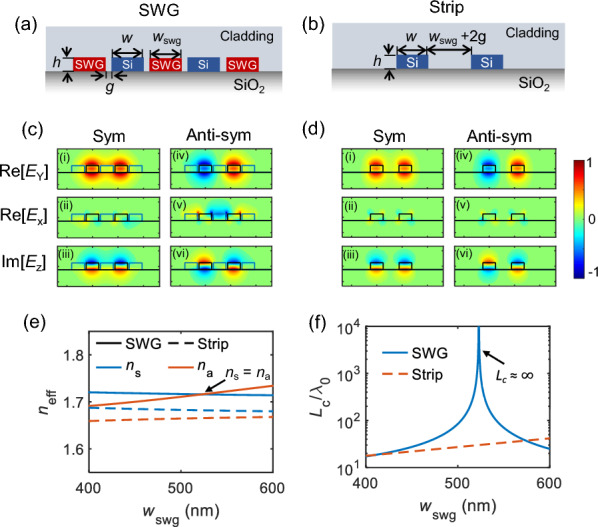
Modal characteristics of the coupled SWG waveguides with the zero-crosstalk singularity. Schematic cross-sections of the coupled **a** SWG and **b** strip waveguides. Electric field profiles of the coupled **c** SWG and **d** strip waveguides: (i–iii) symmetric (sym) and (iv–vi) anti-symmetric (anti-sym) modes. **e** Simulated effective indices of the coupled symmetric ($\textit{n}_{\text{s}}$, blue) and anti-symmetric ($\textit{n}_{\text{a}}$, orange) modes for SWG (solid line) and strip (dashed line) waveguide configurations. The TM modes are examined in both configurations. Note the index crossing point ($\textit{n}_{\text{s}}$ = $\textit{n}_{\text{a}}$) for the SWG case (indicated by arrow). **f** The corresponding normalized coupling lengths ($\textit{L}_{\text{c}}/\lambda _{\text{0}}$) for SWG (blue solid) and strip (orange dashed) waveguides. The arrow indicated the same index crossing point, i.e., the zero-crosstalk singularity where the coupling length goes to infinity $\textit{L}_{\text{c}}$ $\approx \infty$. Geometric parameters are $\textit{h}=220$ nm, $\textit{w}=500$ nm, and $\textit{g}=65$ nm. The free space wavelength is $\lambda _{\text{0}}=1550$ nm, and the cladding index is $\textit{n}_{\text{clad}}=1.33$

Figure 2a shows the cross-sections of the coupled SWG waveguides with cladding index $\textit{n}_{\text{clad}}$ = 1.33, assuming water. Figure 2b is a similar schematic of the coupled strip waveguides without SWG cladding for comparison. The geometric parameters are the same as described in Fig. 1, and the values of periodicity $\Lambda$, fill fraction $\rho$, and gap $\textit{g}$ are chosen based on the feature size, which can be reliably achieved by the e-beam lithography and the subsequent etching process. Figure 2c and d show the numerically simulated (Lumerical Mode solver) electric field components Re[$\textit{E}_{\text{y}}$], Re[$\textit{E}_{\text{x}}$], and Im[$\textit{E}_{\text{z}}$] of the coupled symmetric and anti-symmetric modes for TM polarization: (c) SWG and (d) strip waveguides. The fields in the SWG claddings experience leaky-like oscillations and exhibit non-negligible field components $\textit{E}_{\text{x}}$ and $\textit{E}_{\text{z}}$ [43]. These non-negligible field components are due to the quasi-nature of the TM mode, whose $\textit{E}_\text{x}$ and $\textit{E}_{\text{z}}$ components are enhanced due to the anisotropic nature of SWGs, critical to achieving the zero crosstalk. The leaky-like oscillations in the SWG cladding and the enhanced fields at the gap region can increase the device’s sensitivity, which will be discussed later.

Figure 2e shows the simulated effective refractive indices of symmetric ($\textit{n}_{\text{s}}$, blue) and anti-symmetric ($\textit{n}_{\text{a}}$, orange) modes. The solid and dashed lines indicate the coupled SWG and strip waveguides, respectively, as a function of the SWG $\textit{w}_{\text{swg}}$ that is proportional to the separation distance between the two waveguide channels. The free space wavelength is $\lambda _{\text{0}}$ = 1550 nm, and the TM polarization is examined. Based on the simulated $\textit{n}_{\text{s}}$ and $\textit{n}_{\text{a}}$, we can extract the coupling length $\textit{L}_{\text{c}}$, which is the length required to transfer power from one waveguide to another. In the case of DC, the total device length $\textit{L}$ needs to be matched with this coupling length for an optimal power transfer, but in our case, it is not required since we are utilizing the zero-crosstalk response that is free from the device length *L*. Here, we quantify this $\textit{L}_{\text{c}}$ for examining the degree of crosstalk, not for coupling length matching. The normalized coupling length $\textit{L}_{\text{c}}/\lambda _{\text{0}}$ can be calculated by the index difference $\Delta n$ between $\textit{n}_{\text{s}}$ and $\textit{n}_{\text{a}}$, and can be expressed as [42]

2 $$\begin{aligned} \frac{L_{\text{c}}}{\lambda _0} = \frac{1}{2\mid \!\Delta n\!\mid }=\frac{1}{2\mid \! n_{\text{s}}-n_{\text{a}}\!\mid }. \end{aligned}$$

The coupling length is a good indicator for quantifying the degree of crosstalk for the given coupled modes, as it is independent of the actual device length. Note that, in Fig. 2e, the indices $\textit{n}_{\text{s}}$ and $\textit{n}_{\text{a}}$ of the coupled SWG waveguides cross at a certain $\textit{w}_{swg}\approx 525$ nm, achieving the index difference $\Delta n=0$ (solid lines in Fig. 2e). From Eq. (2), this corresponds to the infinitely long coupling length, i.e., $\textit{L}_{\text{c}}/\lambda _{\text{0}}$ =$\infty$ as clearly shown in Fig. 2f. On the other hand, for the strip waveguide case, $\textit{n}_{\text{s}}$ is always higher than $\textit{n}_{\text{a}}$ (i.e., $\textit{n}_\text{s}>\textit{n}_{\text{a}}$), having finite coupling length $\textit{L}_{\text{c}}/\lambda _{\text{0}}<100$ that is trivial in typical DCs (dashed lines in Fig. 2e). The crosstalk or output power ratio between the coupled ($\textit{I}_{\text{C}}$) and through ($\textit{I}_{\text{T}}$) ports can be expressed as [42],

3 $$\begin{aligned} \textit{Power ratio}=\frac{I_{\text{C}}}{I_{\text{T}}} = \tan ^{2}{\left( \frac{\pi L}{2\textit{L}_{\text{c}}} \right) }. \end{aligned}$$

According to Eq. (3), at the point where $\textit{n}_\text{s}=\textit{n}_{\text{a}}$ (i.e., $\textit{L}_{\text{c}}/\lambda _\text{0}=\infty$), the coupled and through port power ratio approaches zero ($\textit{I}_{\text{C}}/\textit{I}_{\text{T}}=0$), thus labeled as zero power (ZP) point onwards. Again, we are utilizing the ZP point for sensing, which is distinctive from generic DCs that require $\textit{L}=\textit{L}_{\text{c}}$ condition for max power transfer. In our ZP point, $\textit{L}_{\text{c}}$ goes to infinity only with cross-sectional parameters, thus achieving it independent of the device length. In other words, we can choose a device length less than the coupling length to generate a spectral dip in the transmission spectrum of the RI sensor.

### Design optimization for 3D scheme

**Fig. 3:**
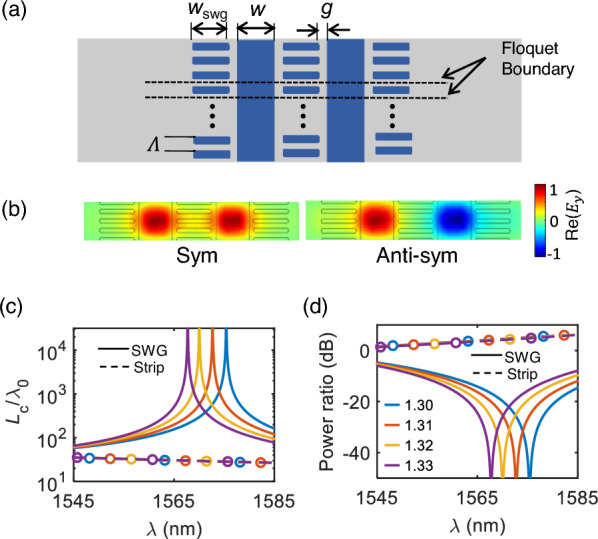
Design optimization with 3D Floquet modal simulations. **a** Top view of the simulation domain. The structure is periodically repeated in the propagation direction (*z*-axis) with Floquet boundary conditions. Geometric parameters are set as $\Lambda =100$ nm, $\rho =0.5$, $w=500$ nm, $w_{\text{swg}}=500$ nm, and $g=65$ nm. **b** Mode profiles of the simulated symmetric and anti-symmetric modes. **c** Simulated normalized coupling length $\textit{L}_{\text{c}}/\lambda$ for different cladding indices for SWG (solid) and strip (dashed circle): $\textit{n}_{\text{clad}}=$1.30 (blue), 1.31 (red), 1.32 (yellow), and 1.33 (purple). **d** Corresponding power ratio for the device length of $L=30$ μm

We then optimized the structures with practical parameters to demonstrate the proposed coupled SWGs-based sensing scheme. It should be noted that all the numerical analyses in the previous section were performed using the effective medium theory, which provides a good insight but would have slight variations in geometric parameters in practice. For the optimization, we used 3D Floquet modal simulation in COMSOL. Figure 3a shows the top view of the simulation domain, where Floquet boundary conditions were imposed on both the top and bottom along the propagation direction. Floquet periodicity was set by defining the wavevector as $\textit{k}_\text{z}=2\pi \textit{n}_{\text{eff}}/\lambda$ where $\textit{n}_{\text{eff}}$ is the effective index of the coupled $\hbox {TM}_{0}$ modes. Subsequently, we determined their eigenfrequencies through numerical simulations. This process was repeated while tracking the coupled $\hbox {TM}_{0}$ symmetric and anti-symmetric modes (Fig. 3b). After tracking the $\textit{n}_{\text{s}}$ and $\textit{n}_{\text{a}}$ of the practical SWG scheme in Fig. 3a, we calculated the coupling length $\textit{L}_{\text{c}}$ using Eq. (2). The solid lines in Fig. 3c show simulated normalized coupling length $\textit{L}_{\text{c}}/\lambda _{\text{0}}$ as a function of the wavelength. Different colors represent different cladding indices: $\textit{n}_{\text{c}}$=1.30 (blue), 1.31 (red), 1.32 (yellow), and 1.33 (purple). Then the expected output power ratio $\textit{I}_{\text{C}}$/$\textit{I}_{\text{T}}$ can be calculated using Eq. (3). Figure 3d shows the corresponding simulated power ratios for each cladding index $\textit{n}_{\text{c}}$. Here, the physical device length is set to $\textit{L}=30$ $\mu$m, considering $L<{L}_{\text{c}}$. In Fig. 3c and d, circle-dashed lines represent the case of conventional strip waveguides without SWGs, and different colors indicate each cladding index. These four circle-dashed lines are indistinguishable, as noted in the previous section, due to the negligible change in $\Delta n = (n_{\text{s}}-n_{\text{a}})$ despite the variations in $\textit{n}_{\text{s}}$ and $\textit{n}_{\text{s}}$ themselves. However, for the coupled SWG cases (solid lines), the results in Fig. 3c and d clearly demonstrate the shift of the ZP wavelength as the cladding index changes.

### Sensitivity enhancement with SWG

The effect of SWGs on sensor performance was analyzed by calculating the device sensitivity $\textit{S}$. The $\textit{S}$ represents the rate of change of the ZP wavelength with respect to the cladding index change. By utilizing Eq. (2) and taking a small cladding index variation $\partial n_{\text{c}}$ to ZP wavelength [46],

4 $$\begin{aligned} S&=\frac{\partial \lambda }{\partial n_{\text{c}}}=\frac{\partial L_{\text{c}}/\partial n_{\text{c}}}{\partial \textit{L}_{\text{c}}/\partial \lambda } \nonumber \\&=\frac{-\lambda }{(n_{\text{s}}-n_{\text{a}})-\lambda ({\partial n_\text{s}}/{\partial \lambda }-{\partial n_{\text{a}}}/{\partial \lambda })}\left( \frac{\partial n_{s}}{\partial n_{c}}-\frac{\partial n_{a}}{\partial n_{c}}\right) . \end{aligned}$$

Then, the magnitude of device sensitivity |*S*| can be represented by

5 $$\begin{aligned} |S|=\lambda \frac{|\Delta S_{\text{m}}|}{|\Delta n_\text{g}|}, \end{aligned}$$

where $\Delta S_{\text{m}}$ is the modal sensitivity difference, defined by $\Delta S_{\text{m}}=S_{\text{sym}}-S_{\text{anti}}=\frac{\partial n_{s}}{\partial n_{c}}-\frac{\partial n_{a}}{\partial n_{c}}$, and $\Delta n_{\text{g}}$ is the group index difference between symmetric and anti-symmetric modes, $\Delta n_{\text{g}}={n_{\text{g}}^\text{sym}}-{n_{\text{g}}^{\text{anti}}}$. Note that the device sensitivity *S* is contingent upon the modal sensitivities ($S_{\text{sym}}$ and $S_{\text{anti}}$) and group index difference $\Delta n_{\text{g}}$. The modal sensitivity is defined as the change in the effective index of the mode in response to the cladding index change and is directly related to the fraction of the electric field intensity in the cladding [47, 48]. The external confinement factor $\Gamma _{\text{clad}}$, which signifies the fraction of electric field intensity in the cladding, is where the optical mode interacts with the sensing analyte volume and can be defined as [47, 48],

6 $$\begin{aligned} \Gamma _{\text{clad}} = \frac{\iiint _{\text{clad}}|{\text{E}}|^2\,dx\,dy\,dz}{\iiint _{\infty }|{\text{E}}|^2\,dx\,dy\,dz}. \end{aligned}$$

**Fig. 4:**
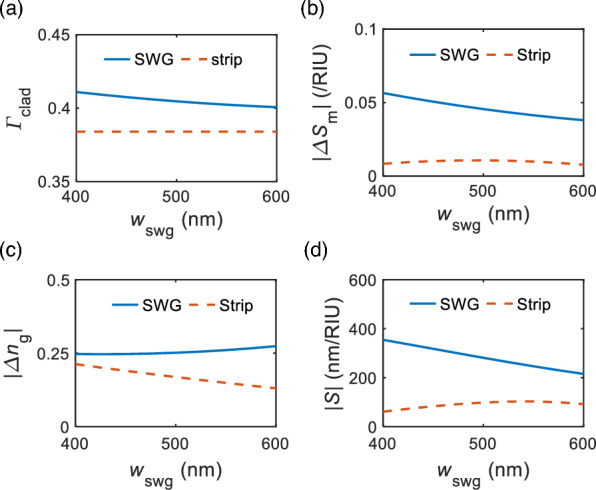
Sensitivity analysis of the coupled SWG and strip waveguides. **a** External confinement factor in the cladding ($\Gamma _{\text{clad}}$) for a single waveguide with SWG claddings (blue) and single strip waveguide (orange). Simulated **b** Modal sensitivity difference $|\Delta S_{\text{m}}|$ and **c** Group index difference between the symmetric and anti-symmetric modes ($|\Delta n_{\text{g}}|$). **d** Calculated device sensitivity |*S*| (nm/RIU) of the coupled SWG (blue) and strip (orange) configurations using Eq. (5). Geometric parameters are set as $\Lambda =100$ nm, $\rho =0.5$, $\textit{w}=500$ nm, and $\textit{g}=65$ nm. The free space wavelength is $\lambda _{0}=1550$ nm

Figure 4a shows the simulated external confinement factor from the 3D Floquet modal simulations. A non-coupled single waveguide configuration is examined with (solid blue) and without (dashed orange) SWG cladding. For calculating the $\Gamma _{\text{clad}}$, we used Eq. (6) while defining the water index region as a cladding. For the geometric parameters, the SWG width is varied for $\textit{w}_{\text{swg}}=400-600$ nm while fixing the other parameters the same as in Fig. 3. It is clearly shown that the external confinement factor of the SWG waveguide is higher than that of the conventional strip waveguide, suggesting a higher RI sensitivity with the SWG configuration. This higher external confinement factor with the SWG waveguide is due to lower field confinement in the core, having leaky mode-like field oscillations in the SWG claddings [43].

The modal sensitivities of our device were accessed by calculating ${\partial n_{\text{eff}}}/{\partial \textit{n}_{\text{c}}}$ for cladding index changes from 1.30 to 1.34. As noted in Eq. (5), the magnitude of device sensitivity $|\textit{S}|$ is proportional to the difference in modal sensitivities between symmetric and anti-symmetric modes (i.e., $|\Delta S_{\text{m}}|=|S_{\text{sym}}-S_\text{anti}|=|{\partial n_{s}}/{\partial n_{c}}-{\partial n_{a}}/{\partial n_{c}}|$) and is inversely proportional to the group index difference (i.e., $|\Delta n_{\text{g}}|=|{n_{\text{g}}^{sym}}-{n_\text{g}^{anti}}|$). For this, we first simulated the modal sensitivities and group indices of both symmetric and anti-symmetric modes (see supplementary S1). We then calculated the modal sensitivity difference $|\Delta S_{\text{m}}|$ and the group index difference $|\Delta n_{\text{g}}|$, as a function of $\textit{w}_{\text{swg}}$ shown in Fig. 4b and c, respectively. Using these data, we determined the total device sensitivity $|\textit{S}|$. The modal sensitivity difference $|\Delta S_{\text{m}}|$ for SWG is approximately four times higher than that of the strip case (Fig. 4b), evidently due to the higher external confinement factor with SWGs. However, for the group index difference, both remain comparably scaled, leading to $|\textit{S}|$ primarily being dominated by the modal sensitivity difference (the group index difference of SWG is slightly higher than that of the strip). This is more directly shown from the calculated device sensitivity in Fig. 4d. Our calculations suggest that the SWG-based device has a spectral sensitivity of around 290 nm/RIU, i.e., substantially surpassing the sensitivity of traditional strip-based DC configuration (approximately 90 nm/RIU compared at $\textit{w}_{\text{swg}}=500$ nm case).

## Experimental results and discussion

### Fabrication and characterization

After optimizing the structure, we fabricated the devices on a 220 nm thick SOI wafer with a 2 μm $\hbox {SiO}_2$ box. Electron beam lithography (JEOL system with 100 keV) was used to pattern the HSQ (Hydrogen silsesquioxane) resist, followed by etching with $\hbox {Cl}_2$/$\hbox {O}_2$ gas.

**Fig. 5:**
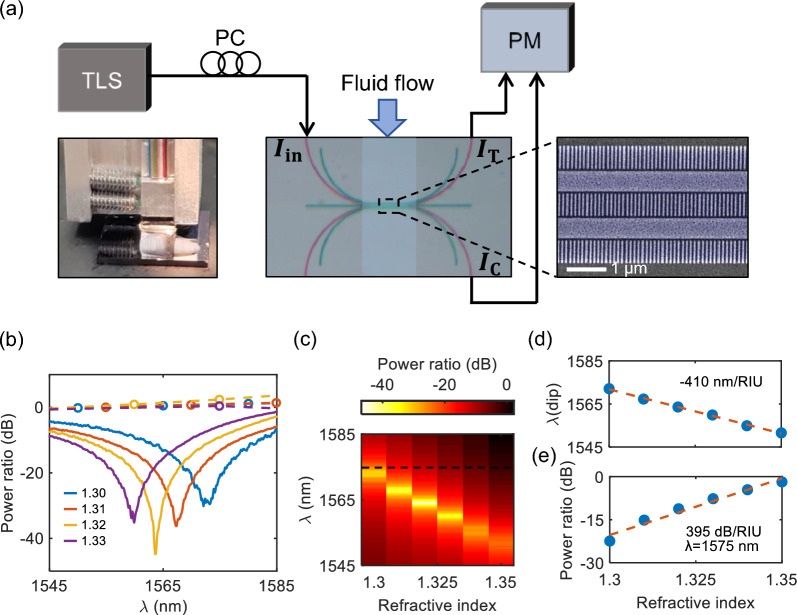
Experimental demonstration of RI sensing in coupled SWG waveguides. **a** Experimental characterization setup with optical image and SEM image of the device **b** Measured power ratio of the strip (dashed circle) and SWG (solid) waveguides for RI sensing with the cladding liquid indices 1.30 (blue), 1.31 (red), 1.32 (yellow), and 1.33 (purple). **c** Map plot of the transmission spectra for cladding index changes from 1.30 to 1.35. The dashed black line indicates the wavelength at $\lambda =1575$ nm for **e**. **d** Spectral dip shift and **e**) Power ariation at the fixed wavelength of $\lambda =1575$ nm, while varying the cladding indices from 1.3 to 1.35. The blue circles indicate measured values, and the orange dashed lines are their linear fits. The characterized spectral and intensity sensitivities are approximately $-410\pm 0.5$ nm/RIU and $395\pm 1.9$ dB/RIU, respectively

Figure 5a illustrates the experimental setup designed to evaluate the sensing performance of our devices. Arrows coming in and out of the optical image of the device in Fig. 5a represent the power input ($\textit{I}_{\text{in}}$) and output ($\textit{I}_{\text{T}}$ and $\textit{I}_\text{C}$) ports. We employed grating couplers for fiber-to-chip couplings, specifically designed for $\hbox {TM}_{0}$ mode excitation with a 15-degree angle-polished optical fiber array. The SWG structures were extended and bent to facilitate the adiabatic modal transition from the strip to the SWG region [42, 43]. The SEM image on the right shows the zoomed-in coupling region, detailing our devices with the SWG structures.

For spectral response measurement, we utilized a tunable laser source (TLS) (Keysight 81608A). The input light’s polarization was controlled using a manual fiber polarization controller (PC). The out-coupled signals were sent to a multichannel optical power meter (PM) (Keysight N7744A), which simultaneously measured the powers in the through and coupled ports, denoted as $\textit{I}_{\text{T}}$ and $\textit{I}_{\text{C}}$, respectively. The power ratio spectra measured in this system can be used for calculating coupling length using Eq. (3). To ascertain device sensitivity, we applied standard refractive index liquids from Cargille labs to the cladding area of the device. These liquids were carefully applied to the device’s cladding area to ensure complete coverage, as shown in the top optical image of the experimental setup in Fig. 5a. Figure 5b shows the experimentally measured power-ratio spectra in response to cladding index changes from 1.30 to 1.33. Each color denotes different cladding indices, as previously shown in Fig. 3d. As expected from Fig. 3d, an evident blue shift in the spectral dip is observed as the cladding index increases. The degree of shift is slightly larger than our numerical findings, a deviation possibly attributed to fabrication imperfections affecting the filling fraction in our realized SWGs compared to simulations. It is noteworthy that the periodicity of our SWGs is 100 nm, and 5 nm errors can induce a 5$\%$ change in the filling fraction. A reduced Si filling fraction could enhance the cladding index’s impact on the spectral shift, as explained in supplementary S2. Figure 5c shows the map plot of the power ratio for all the cladding index changes from 1.3 to 1.35. The bright spots in this map plot indicate the position of the zero power ratio point.

Utilizing these spectral results, we then characterized the wavelength and power sensitivities. Figure 5d summarizes the wavelengths of the ZP ratio point for each cladding refractive index, representing wavelength sensitivity. A linear fit denoted by the red dashed line estimates the wavelength sensitivity of approximately $-410\pm 0.5$ nm/RIU. The negative sign represents the blue shift, with the uncertainty based on one standard deviation. This negative sensitivity with the blue shift is also a unique spectral characteristic of our zero-crosstalk-based sensor that is opposite to most of the previous spectral responses. The characterized sensitivity is slightly higher than the numerical results in Section 2, probably due to less filling fraction of SWG during the fabrication process (see Supplementary Fig. S2). The map plot in Fig. 5c also illustrates the power ratio shift for each wavelength, which is valuable for intensity sensing. For example, by fixing the wavelength at $\lambda =1575$ nm (along the black dashed line in Fig. 5c), we could plot the power ratio across cladding index changes, as shown in Fig. 5e. The linear fit (orange dashed line) yields a power sensitivity of approximately $395\pm 1.9$ dB/RIU. It is worth noting that this sensitivity should be significantly higher near the zero-ratio wavelength, as explained in Supplementary S3.

### Real-time intensity sensing

**Fig. 6:**
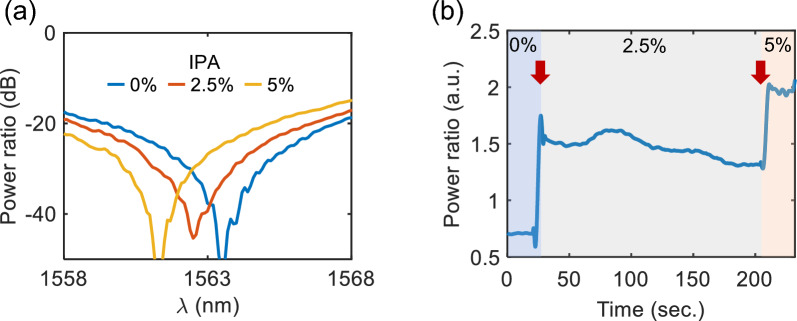
Real-time sensing with different IPA concentrations in DI water. **a** Spectral responses when IPA concentrations are $0\%$ (blue), $2.5\%$ (orange), and $5\%$ (yellow). **b** Time-domain response evolution. Red arrows indicate the dropping points. The output power ratio variations are recorded as the droplets are being added: light blue, grey, and pink area denotes $0\%$, $2.5\%$, and $5\%$ IPA concentrations, respectively

For an ideal RI sensor, the capability for real-time monitoring is essential. Intensity sensing in real-time is particularly attractive since it eliminates the need for a tunable laser source. To assess the suitability of our scheme for real-time RI sensing, we used DI water mixed with different concentrations of IPA (Isopropyl Alcohol) solutions instead of refractive index liquids. These solutions are expected to change the water refractive index at a rate of $\approx$0.0008 RIU/percent [48]. Following a similar procedure as in Fig. 5, we first examined the power-ratio spectra corresponding to each IPA concentration, shown in Fig. 6a. The IPA concentrations were 0$\%$ (blue), 2.5$\%$ (red), and 5.0$\%$ (yellow). Analogous to Fig. 5b, a clear blue shift was observed with increasing the IPA concentration. By comparing these spectral results with Fig. 5d, the refractive indices of 0$\%$, 2.5$\%$, and 5.0$\%$ IPA concentrations can be estimated to be approximately 1.333, 1.335, and 1.337, respectively.

Subsequently, we conducted real-time monitoring. We fixed the input laser wavelength at 1565 nm and continuously monitored the output power ratio while sequentially adding different concentrations of IPAs (Fig. 6b). The initial measurement was taken with $0\%$ IPA water, followed by adding $2.5\%$ and $5.0\%$ IPA solutions at $\approx 20$ s and $\approx 210$ s, respectively (as marked by the red arrows). For the sake of simplicity, we dropped each subsequent IPA concentration on top of the preceding one. This resulted in almost immediate observable changes in the power level. The slight irregularities in the signal are likely due to the fluctuating refractive index profile near the waveguides, caused by the diffusion and blending of liquids with different concentrations. Employing a more meticulous and precise methodology for sample handling, like using a microfluidic channel, could enhance the quality of the signal, yielding a clearer and more sensitive temporal evolution.

### System limit of detection

The system limit of detection ($\textit{sLOD}$) determines the minimum refractive index change measurable by the sensor. This metric depends on the device’s sensitivity *S* and the noise level of the detection system. The $\textit{sLOD}$ is given by [9, 12],

7 $$\begin{aligned} {\textit{s}LOD} = {\frac{3\sigma }{S}} \end{aligned}$$

where $\sigma$ represents the total system noise. To estimate the total system noise, we repeated sampling of the sensor’s spectrum. For wavelength sensing, we calculated the standard deviation of the measured ZP-ratio wavelength from 20 measurements ($\sigma _{\lambda }=0.031$ nm). Similarly, we also calculated the power deviation in those 20 sampling spectra and calculated the standard deviation of the measured power ($\sigma _{P}=0.068$ dB). See supplementary S4 for further details on measuring $\sigma _{\lambda }$ and $\sigma _{P}$. Then, using the measured wavelength sensitivity of $-410$ nm/RIU, the wavelength system limit of detection $sLOD_{\lambda }$ was found to be $\approx 2.4\times 10^{-4}$ RIU. Following the same procedure and using the intensity sensitivity of 395 dB/RIU, the intensity system limit of detection $sLOD_{I}$ was determined to be $\approx 5.2\times 10^{-4}$ RIU (at $\lambda =1575$ nm).

### Performance comparison

Table 1 summarizes a comprehensive overview of high-performance silicon photonic RI sensors, emphasizing spectral sensitivity, their suitability for fixed wavelength intensity sensing, and the sensor’s footprint. Given that most literature mainly reports spectral sensitivity, a direct intensity sensitivity comparison is challenging. Thus, we have employed tick marks ($\checkmark$) to signify the relative strengths of each sensor configuration in terms of intensity sensitivity.

**Table 1:** Summary of silicon photonic RI sensors

Device	Mechanism	*S* (nm/RIU)	FSR	Intensity sensitivity^a^	LOD	Footprint *F* (μm^2^)	*S*/*F* (nm/RIU/μm^2^)
MZI [18]	Interference	2870	0.991 nm	$\checkmark \checkmark$	–	6.25 $\times 10^{4}$	0.04
Bragg [21]	Resonance	340	Limited	$\checkmark$	3 $\times 10^{-4}$	92	3.69
PhC [49]	Resonance	340	Free	$\checkmark$	2.78 $\times 10^{-4}$	11	38.90
PhC [37]	Resonance	586	Free	$\checkmark$	6.29 $\times 10^{-4}$	34	17.2
SWG Ring [35]	Resonance	490	3.93 nm	$\checkmark$	2 $\times 10^{-6}$	706	0.69
SWG Ring [50]	Resonance	440	12.5 nm	$\checkmark$	3.9 $\times 10^{-4}$	314	1.4
SWG Racetrack [36]	Resonance	430	13 nm	$\checkmark$	3.7 $\times 10^{-4}$	424	1.01
SWG Bragg [39]	Resonance	579	Limited	$\checkmark$	$10^{-6}$	228	2.53
SWG Bimodal [40]	Interference	2270	20/60 nm	$\checkmark \checkmark$	–	175	12.9
**This work**	**Zero crosstalk**	**410**	**Free**	$\checkmark \checkmark \checkmark$	**2.4** $\times 10^{-4}$	**82.8**	**4.95**

Bold text highlights this work

S, Spectral sensitivity; FSR, Free-spectral range; MZI, Mach–Zehnder interferometer; SWG, Subwavelength grating; LOD, Limit of detection; PhC, Photonic crystal)

^a^Tick marks ($\checkmark$) indicate the relative strength of suitability to perform intensity sensing

In terms of the spectral sensitivity *S* per footprint (nm/RIU/μm^2^) comparison, our device outperforms most of the other devices except for the PhC [37, 49] and SWG bimodal sensors [40]. Although PhC sensors are FSR-free and require less footprint, their Lorentzian resonance complicates intensity interrogation over a wide RI range (similar to ring resonator sensors). Thus, in Table 1, a single tick was given to this type in the suitability column of intensity interrogation. For the MZI and SWG bimodal sensors, sinusoidal responses favor intensity sensing, but a limited FSR can restrict the detection range and introduce potential ambiguity. Consequently, the MZI and bimodal sensors are marked with double ticks. Here, our SWG sensor distinguishes itself with an FSR-free operation, allowing for an extensive detection range, and its tangential nature of the spectrum facilitates simple intensity readings at fixed wavelengths, thus earning three ticks in comparison to others. The $\textit{sLOD}$ of our device is comparable to those reported ones, indicating its viability to perform sensitive sensing in both wavelength and intensity schemes. It’s worth mentioning that the precision of this $\textit{sLOD}$ can be enhanced with more refined sample handling methods, like microfluidic setup and using balanced detection for intensity sensing. In comparing the sensing footprint, our device has the smallest size among sensors capable of both wavelength and intensity sensing.

## Conclusion

In conclusion, we have successfully demonstrated an ultracompact silicon photonic refractive index sensor utilizing unique zero-crosstalk responses enabled by SWG anisotropic metamaterials. We successfully designed and achieved zero-crosstalk responses near telecommunication wavelengths, enabling both wavelength and intensity interrogations. Our experimental results show a wavelength sensitivity of $-410~\text {nm/RIU}$ and an intensity sensitivity of $395~\text {dB/RIU}$. The designed device length was set at 30 μm, resulting in a compact total footprint of approximately 82.8 μm^2^. Our approach of incorporating SWG in the cladding region enhances external confinement factors, significantly increasing the device’s sensitivity compared to traditional DC-based RI sensors. The single-dip spectral response of our approach enables FSR-free operation with a broad detection range, while the tangential-shaped spectral response facilitates easy mapping with refractive index variations. The simplicity of our device architecture, combined with its high sensitivity per unit area, makes it highly suitable for intensity interrogation. Thus, our device holds great promise for on-chip RI sensing applications, such as gas or biochemical sensing.

## Supplementary Information

Supplementary Material 1.

## Data Availability

All data generated or analyzed during this study are included in this published article and its supplementary information files.

## Abbreviations

RI: Refractive index
SWG: Subwavelength grating
MZI: Mach–Zehnder interferometer
PhC: Photonic crystal
FSR: Free spectral range
DC: Directional coupler
SOI: Silicon-on-insulator
EMT: Effective medium theory
Si: Silicon
ZP: Zero power
HSQ: Hydrogen silsesquioxane
TLS: Tunable laser source
PC: Polarization controller
PM: Power meter
IPA: Isopropyl alcohol
sLOD: System limit of detection

## References

[1] S.Y. Siew, B. Li, F. Gao, H.Y. Zheng, W. Zhang, P. Guo, S.W. Xie, A. Song, B. Dong, L.W. Luo, C. Li, X. Luo, G.Q. Lo, Review of silicon photonics technology and platform development. J. Lightwave Technol. **39**(13), 4374–4389 (2021)

[2] W. Shi, Y. Tian, A. Gervais, Scaling capacity of fiber-optic transmission systems via silicon photonics. Nanophotonics **9**(16), 4629–4663 (2020)

[3] B.J. Shastri, A.N. Tait, T. Ferreira de Lima, W.H. Pernice, H. Bhaskaran, C.D. Wright, P.R. Prucnal, Photonics for artificial intelligence and neuromorphic computing. Nat. Photonics **15**(2), 102–114 (2021)

[4] L. Feng, M. Zhang, J. Wang, X. Zhou, X. Qiang, G. Guo, X. Ren, Silicon photonic devices for scalable quantum information applications. Photonics Res. **10**(10), A135–A153 (2022)

[5] D. Jeon, K. Shin, S.W. Moon, J. Rho, Recent advancements of metalenses for functional imaging. Nano Converg. **10**(24), 5 (2023) 37222959 10.1186/s40580-023-00372-8 PMC10209387

[6] Z. Zhang, Y. Lee, M.F. Haque, J. Leem, E.Y. Hsieh, S. Nam, Plasmonic sensors based on graphene and graphene hybrid materials. Nano Converg. **9**(28), 1–24 (2022) 35695997 10.1186/s40580-022-00319-5 PMC9192873

[7] H. Kim, H.J. An, J. Park, Y. Lee, M.S. Kim, S. Lee, N.D. Kim, J. Song, I. Cho, Ultrasensitive and real-time optical detection of cellular oxidative stress using graphene-covered tunable plasmonic interfaces. Nano Converg. **9**(23), 1–12 (2022) 35604511 10.1186/s40580-022-00315-9 PMC9127018

[8] L. Kim, S. Jo, G.J. Kim, K.H. Kim, S.E. Seo, E. Ryu, C.J. Shin, Y.K. Kim, J.W. Choi, O.S. Kwon, Recombinant protein embedded liposome on gold nanoparticle based on LSPR method to detect corona virus. Nano Converg. **10**(51), 1–10 (2023) 37902883 10.1186/s40580-023-00399-x PMC10615991

[9] E. Luan, H. Shoman, D.M. Ratner, K.C. Cheung, L. Chrostowski, Silicon photonic biosensors using label-free detection. Sensors **18**(10), 3519 (2018) 30340405 10.3390/s18103519 PMC6210424

[10] N.L. Kazanskiy, S.N. Khonina, M.A. Butt, Advancement in silicon integrated photonics technologies for sensing applications in near-infrared and mid-infrared region: a review. Photonics **9**(5), 331 (2022)

[11] A. Densmore, D.X. Xu, S. Janz, P. Waldron, T. Mischki, G. Lopinski, A. Delâge, J. Lapointe, P. Cheben, B. Lamontagne, J.H. Schmid, Spiral-path high-sensitivity silicon photonic wire molecular sensor with temperature-independent response. Opt. Lett. **33**(6), 596–598 (2008) 18347721 10.1364/ol.33.000596

[12] Q. Liu, X. Tu, K.W. Kim, J.S. Kee, Y. Shin, K. Han, Y.J. Yoon, G.Q. Lo, M.K. Park, Highly sensitive mach-zehnder interferometer biosensor based on silicon nitride slot waveguide. Sens. Actuators, B **188**, 681–688 (2013)

[13] L. Laplatine, M. Fournier, N. Gaignebet, Y. Hou, R. Mathey, C. Herrier, J. Liu, D. Descloux, B. Gautheron, T. Livache, Silicon photonic olfactory sensor based on an array of 64 biofunctionalized mach-zehnder interferometers. Opt. Expr. **30**(19), 33955–33968 (2022) 10.1364/OE.461858 36242419

[14] J.T. Robinson, L. Chen, M. Lipson, On-chip gas detection in silicon optical microcavities. Opt. Express. **16**(6), 4296–4301 (2008) 18542525 10.1364/oe.16.004296

[15] M. Iqbal, M.A. Gleeson, B. Spaugh, F. Tybor, W.G. Gunn, M. Hochberg, T. Baehr-Jones, R.C. Bailey, L.C. Gunn, Label-free biosensor arrays based on silicon ring resonators and high-speed optical scanning instrumentation. IEEE J. Sel. Top. Quantum Electron. **16**(3), 654–661 (2010)

[16] S. Hu, Y. Zhao, K. Qin, S.T. Retterer, I.I. Kravchenko, S.M. Weiss, Enhancing the sensitivity of label-free silicon photonic biosensors through increased probe molecule density. ACS Photonics **1**(7), 590–597 (2014)

[17] Y. Lee, H.F. Zhang, C. Sun, Highly sensitive ultrasound detection using nanofabricated polymer micro-ring resonators. Nano Converg. **10**(30), 1–19 (2023) 37338745 10.1186/s40580-023-00378-2 PMC10281933

[18] X. Jiang, Y. Chen, F. Yu, L. Tang, M. Li, J.J. He, High-sensitivity optical biosensor based on cascaded Mach-Zehnder interferometer and ring resonator using vernier effect. Opt. Lett. **39**(22), 6363–6366 (2014) 25490469 10.1364/OL.39.006363

[19] H. Zhu, Y. Yue, Y. Wang, M. Zhang, L. Shao, J. He, M. Li, High-sensitivity optical sensors based on cascaded reflective MZIS and microring resonators. Opt. Express. **25**(23), 28612–28618 (2017)

[20] Z. Li, J. Zou, H. Zhu, B.T.T. Nguyen, Y. Shi, P.Y. Liu, R.C. Bailey, J. Zhou, H. Wang, Z. Yang, Y. Jin, P.H. Yap, H. Cai, Y. Hao, A.Q. Liu, Biotoxoid photonic sensors with temperature insensitivity using a cascade of ring resonator and Mach-Zehnder interferometer. ACS Sens. **5**(8), 2448–2456 (2020) 32666782 10.1021/acssensors.0c00622

[21] X. Wang, J. Flueckiger, S. Schmidt, S. Grist, S.T. Fard, J. Kirk, M. Doerfler, K.C. Cheung, D.M. Ratner, L. Chrostowski, A silicon photonic biosensor using phase-shifted BRAGG gratings in slot waveguide. J. Biophotonics **6**(10), 821–828 (2013) 23576430 10.1002/jbio.201300012

[22] H. Li, Z. Zhu, W. Meng, L. Cao, Y. Wang, Z. Lin, E. Li, J.D. Prades, Silicon-photonics-based waveguide bragg grating sensor for blood glucose monitoring. Opt. Express. **30**(23), 41554–41566 (2022) 36366630 10.1364/OE.472137

[23] K. Yao, Y. Shi, High-q width modulated photonic crystal stack mode-gap cavity and its application to refractive index sensing. Opt. Express. **20**(24), 27039–27044 (2012) 23187559 10.1364/OE.20.027039

[24] D. Yang, S. Kita, F. Liang, C. Wang, H. Tian, Y. Ji, M. Lončar, Q. Quan, High sensitivity and high q-factor nanoslotted parallel quadrabeam photonic crystal cavity for real-time and label-free sensing. Appl. Phys. Lett. **105**(6), 063118 (2014)

[25] R. Heideman, P. Lambeck, Remote opto-chemical sensing with extreme sensitivity: design, fabrication and performance of a pigtailed integrated optical phase-modulated mach-zehnder interferometer system. Sens. Actuators, B **61**(1–3), 100–127 (1999)

[26] Y.E. Marin, V. Toccafondo, P. Velha, S. Scarano, S. Tirelli, A. Nottola, Y. Jeong, H. Jeon, S. Kim, M. Minunni, F. Di Pasquale, C.J. Oton, Silicon photonic biochemical sensor on chip based on interferometry and phase-generated-carrier demodulation. IEEE J. Sel. Top. Quantum Electron. **25**(1), 1–9 (2018)

[27] B. Sepúlveda, G. Armelles, L.M. Lechuga, Magneto-optical phase modulation in integrated Mach-Zehnder interferometric sensors. Sens. Actuators, A **134**(2), 339–347 (2007)

[28] S. Dante, D. Duval, B. Sepúlveda, A.B. González-Guerrero, J.R. Sendra, L.M. Lechuga, All-optical phase modulation for integrated interferometric biosensors. Opt. Express. **20**(7), 7195–7205 (2012) 22453401 10.1364/OE.20.007195

[29] Genalyte, inc. https://www.genalyte.com/home/maverick/. Accessed 1 Nov 2023.

[30] S. Mudumba, S. de Alba, R. Romero, C. Cherwien, A. Wu, J. Wang, M.A. Gleeson, M. Iqbal, R.W. Burlingame, Photonic ring resonance is a versatile platform for performing multiplex immunoassays in real time. J. Immunol. Methods **448**, 34–43 (2017) 28527901 10.1016/j.jim.2017.05.005

[31] X. Ou, B. Tang, P. Zhang, B. Li, F. Sun, R. Liu, K. Huang, L. Xie, Z. Li, Y. Yang, Microring resonator based on polarization multiplexing for simultaneous sensing of refractive index and temperature on silicon platform. Opt. Express. **30**(14), 25627–25637 (2022) 36237088 10.1364/OE.459743

[32] B. Luff, R. Harris, J. Wilkinson, R. Wilson, D. Schiffrin, Integrated-optical directional coupler biosensor. Opt. Lett. **21**(8), 618–620 (1996) 19876102 10.1364/ol.21.000618

[33] K. Uchiyamada, K. Okubo, M. Yokokawa, E. Carlen, K. Asakawa, H. Suzuki, Micron scale directional coupler as a transducer for biochemical sensing. Opt. Express. **23**(13), 17156–17168 (2015) 26191724 10.1364/OE.23.017156

[34] K. Okubo, K. Uchiyamada, K. Asakawa, H. Suzuki, Silicon nitride directional coupler interferometer for surface sensing. Opt. Eng. **56**(1), 017101 (2017)

[35] J. Flueckiger, S. Schmidt, V. Donzella, A. Sherwali, D.M. Ratner, L. Chrostowski, K.C. Cheung, Sub-wavelength grating for enhanced ring resonator biosensor. Opt. Express. **24**(14), 15672–15686 (2016) 27410840 10.1364/OE.24.015672

[36] L. Huang, H. Yan, X. Xu, S. Chakravarty, N. Tang, H. Tian, R.T. Chen, Improving the detection limit for on-chip photonic sensors based on subwavelength grating racetrack resonators. Opt. Express. **25**(9), 10527–10535 (2017) 28468425 10.1364/OE.25.010527 PMC5462069

[37] P. Xu, J. Zheng, J. Zhou, Y. Chen, C. Zou, A. Majumdar, Multi-slot photonic crystal cavities for high-sensitivity refractive index sensing. Opt. Express. **27**(3), 3609–3616 (2019) 30732377 10.1364/OE.27.003609 PMC6410913

[38] S.H. Badri, Transmission resonances in silicon subwavelength grating slot waveguide with functional host material for sensing applications. Opt. Laser Technol. **136**, 106776 (2021)

[39] E. Luan, H. Yun, M. Ma, D.M. Ratner, K.C. Cheung, L. Chrostowski, Label-free biosensing with a multi-box sub-wavelength phase-shifted bragg grating waveguide. Biomed. Opt. Express. **10**(9), 4825–4838 (2019) 31565528 10.1364/BOE.10.004825 PMC6757469

[40] L. Torrijos-Morán, A. Griol, J. García-Rupérez, Experimental study of subwavelength grating bimodal waveguides as ultrasensitive interferometric sensors. Opt. Lett. **44**(19), 4702–4705 (2019) 31568421 10.1364/OL.44.004702

[41] S. Jahani, S. Kim, J. Atkinson, J.C. Wirth, F. Kalhor, A.A. Noman, W.D. Newman, P. Shekhar, K. Han, V. Van, R.G. DeCorby, L. Chrostowski, M. Qi, Z. Jacob, Controlling evanescent waves using silicon photonic all-dielectric metamaterials for dense integration. Nat. Commun. **9**(1), 1893 (2018) 29760394 10.1038/s41467-018-04276-8 PMC5951946

[42] M.B. Mia, S.Z. Ahmed, I. Ahmed, Y.J. Lee, M. Qi, S. Kim, Exceptional coupling in photonic anisotropic metamaterials for extremely low waveguide crosstalk. Optica **7**(8), 881–887 (2020)

[43] M.F. Kabir, M.B. Mia, I. Ahmed, N. Jaidye, S.Z. Ahmed, S. Kim, Anisotropic leaky-like perturbation with subwavelength gratings enables zero crosstalk. Light Sci. Appl. **12**(1), 135 (2023) 37268648 10.1038/s41377-023-01184-5 PMC10238401

[44] S.Z. Ahmed, I. Ahmed, M.B. Mia, N. Jaidye, S. Kim, Ultra-high extinction ratio polarization beam splitter with extreme skin-depth waveguide. Opt. Lett. **46**(9), 2164–2167 (2021) 33929444 10.1364/OL.420824

[45] G.W. Milton, *The theory of composites* (Cambridge University Press, 2002)

[46] K. Li, T. Zhang, G. Liu, N. Zhang, M. Zhang, L. Wei, Ultrasensitive optical microfiber coupler based sensors operating near the turning point of effective group index difference. Appl. Phys. Lett. **109**(10), 101101 (2016)

[47] F. Dell’Olio, V.M. Passaro, Optical sensing by optimized silicon slot waveguides. Opt. Express. **15**(8), 4977–4993 (2007) 19532747 10.1364/oe.15.004977

[48] X. Sun, D. Dai, L. Thylén, L. Wosinski, High-sensitivity liquid refractive-index sensor based on a Mach-Zehnder interferometer with a double-slot hybrid plasmonic waveguide. Opt. Express. **23**(20), 25688–25699 (2015) 26480084 10.1364/OE.23.025688

[49] Y. Zhang, S. Han, S. Zhang, P. Liu, Y. Shi, High-q and high-sensitivity photonic crystal cavity sensor. IEEE Photonics J. **7**(5), 1–6 (2015)

[50] H. Yan, L. Huang, X. Xu, S. Chakravarty, N. Tang, H. Tian, R.T. Chen, Unique surface sensing property and enhanced sensitivity in microring resonator biosensors based on subwavelength grating waveguides. Opt. Express. **24**(26), 29724–29733 (2016) 28059356 10.1364/OE.24.029724 PMC5234505

