# Supplementary material for: Zero-crosstalk silicon photonic refractive index sensor with subwavelength gratings
Source: Nano Converg. 2024 Sep 28;11:39. doi: 10.1186/s40580-024-00446-1 (PMC11438762; doi:10.1186/s40580-024-00446-1)
Supplement: Supplementary file 1 — Supplementary Material 1. [file 40580_2024_446_MOESM1_ESM.pdf]

Ultracompact Silicon Photonic Sensor using Waveguide Coupling Singularity with Subwavelength Gratings

Syed Z. Ahmed[1], Mehedi Hasan[1], Kyungtae Kim[2], and Sangsik Kim[1,2,*]

[1]Department of Electrical and Computer Engineering, Texas Tech University, Lubbock, TX 79409
[2]School of Electrical Engineering, Korea Advanced Institute of Science and Technology, Daejeon 34141, South Korea

[*]E-mail: sangsik.kim@kaist.ac.kr

Supplementary Information

S1. Modal sensitivities of SWG and strip waveguides

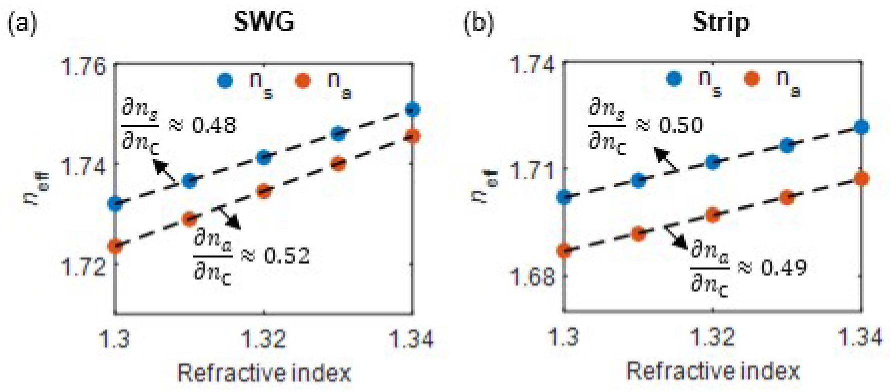

Figure S1: Modal sensitivities of the coupled symmetric and the anti-symmetric mode: coupled (a) SWG and (b) strip waveguides. Blue and orange dots represent the simulated effective indices of the symmetric (n_s) and anti-symmetric (n_a) modes, respectively, while varying the cladding refractive index from 1.30 to 1.34. The black dashed lines are their respective linear fit used to calculate ($\partial n_{eff}/\partial n_c$). The geometrical parameters and the wavelength are the same as in Fig. 4 of the main manuscript. The fitted modal sensitivities are indicated by the black arrow for each case. Due to the leaky nature of SWG mode, the modal sensitivity difference $|\Delta S_m|=|\partial n_s/\partial n_c - \partial n_a/\partial n_c|$ of SWG is approximately 4 times higher than that of strip waveguides.

S2. Device sensitivities with different fill fractions

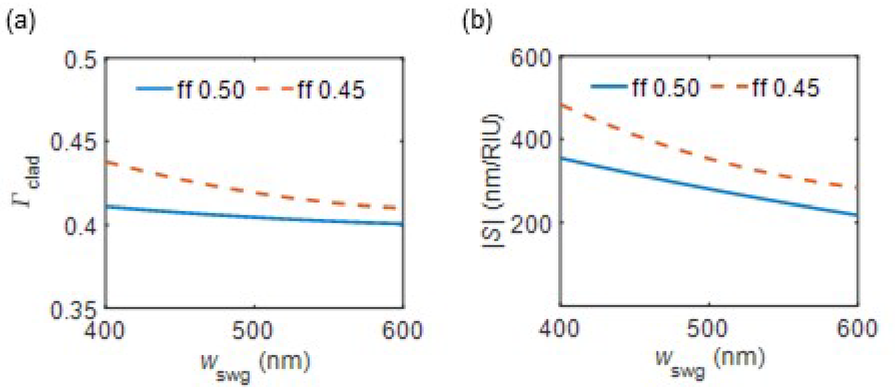

Figure S2: External confinement factor and device sensitivity with different fill fractions. (a) Simulated external confinement factor Γ_{clad} with fill fractions ff=0.50 (solid blue) and 0.45 (dashed orange). Other parameters are the same as in Fig. 4. (a) of the main manuscript. Reducing the fill fraction of SWG increases the external confinement in the cladding. (b) Correspondingly calculated device sensitivities when ff=0.50 (blue solid) and 0.45 (orange dashed). As expected from the increased external confinement factor with a lower filling fraction, a higher sensitivity $|S|$ is observed as the filling fraction decreases (e.g., at w_{swg} =500, $|S| \approx$ 290 nm/RIU for ff=0.5 and ≈350 nm/RIU for ff=0.45.)

S3. Intensity interrogation sensitivity

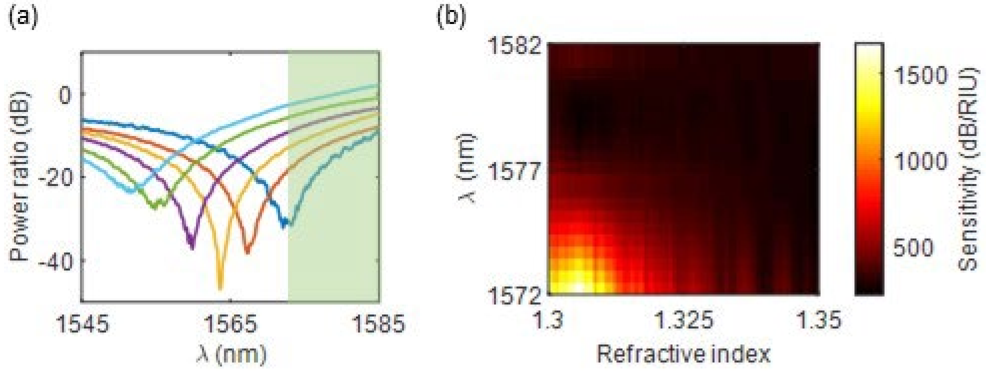

Figure S3. Intensity sensitivity variation with wavelength. (a) Measured spectral data for six different RI index liquids n_c=1.30 (blue), 1.31 (orange), 1.32 (yellow), 1.33 (purple), 1.34 (green), and 1.35 (aqua). (b) Map plot of intensity sensitivity for spectrum after the last dip (the green shaded region). Intensity sensitivity is estimated from the slope of 3rd order polynomial curve fitting of the power ratio at each wavelength in this region.

Figure S3(a) shows the spectral power ratio data when the cladding liquids' refractive indices are n_{clad} = 1.30−1.35. Figure S3(b) is the map plot of the estimated intensity sensitivity of the spectral region after the last spectral dip [green shadow region of Fig. S2(a)]. For a slight change in the index, the power ratio near the spectral dip wavelength will change more rapidly than the other spectral region away from the dip due to the spectrum's tangential nature. Sensitivity away from the spectral dip gives a more linear response in intensity interrogation, which was used to calculate intensity sensitivity shown in Fig. 5(e) of the main manuscript. To show the effect of getting a higher sensitivity near the spectral dip more explicitly, we calculated the map plot of the sensitivity as functions of wavelength and cladding index variation. A third-order polynomial is fitted to the intensity change for each wavelength, starting from the longest spectral dip near λ ≈1572 nm to blue, as shaded with light green in Fig. S3(a). We note much higher intensity sensitivity (≈ 1500 dB/RIU) near the spectral dip at λ ≈1572 nm.

S4. System limit of detection (*sLOD)*

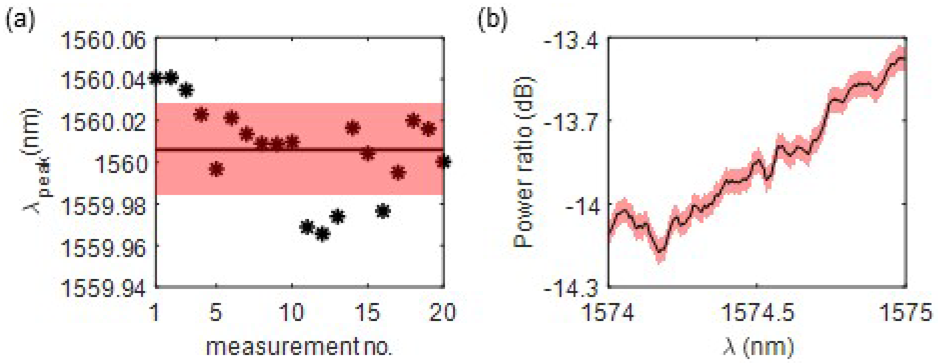

Figure S4. System limit of detection (*sLOD*) characterization. The *sLOD* can be characterized by *sLOD* =3σ/*S*, where σ and *S* are the standard deviation of noise in the system and the device sensitivity, respectively [1]. To characterize this, we repeated the same measurements 20 times and recorded the transmission dips in (a). The black * denotes the minimum wavelength measured, the solid black line indicates the mean value, and the red shaded area represents the ± standard deviation around the mean (σ=0.031 nm). The calculated system limit of detection for the wavelength interrogation is $sLOD_\lambda \approx 2.4\times10^{-4}$ RIU, considering wavelength sensitivity S_λ=410 nm/RIU. Then, a similar approach was repeated for the intensity interrogation by repeated power ratio measurements (20 times). (b) Repeated power ratio measurements: the solid black line indicates the average power for the 1574-1575 nm wavelength range, and the red shaded area represents ± standard power deviation around the mean value (σ=0.068 dB). The calculated intensity system limit of detection is $sLOD_I \approx 5.2\times10^{-4}$ RIU, considering intensity sensitivity S_I=395 dB/RIU (at 1575 nm).

References

[1] Luan, E., Shoman, H., Ratner, D. M., Cheung, K. C., & Chrostowski, L. (2018). Silicon photonic biosensors using label-free detection. *Sensors*, *18*(10), 3519.
